# Real-Time Psychological Factors Using Ecological Momentary Assessment With Continuous Glucose Monitoring in Diabetes Mellitus: Scoping Review

**DOI:** 10.2196/87688

**Published:** 2026-09-30

**Authors:** Kara Mizokami-Stout, Kira Voelker, Melissa DeJonckheere, Lynn Ang, Evan L Reynolds, Joyce M Lee, Rodica Pop-Busui, Brian C Callaghan, Emily Hirschfeld, Jennifer J Iyengar, Dana Albright

**Affiliations:** 1 Division of Metabolism, Endocrinology and Diabetes, University of Michigan, 1000 Wall Street, Ann Arbor, MI, 48105, United States, 1 734-232-1269; 2 Department of Endocrinology and Metabolism, VA Ann Arbor Healthcare System, Ann Arbor, MI, United States; 3 Parkview Mirro Center for Research and Innovation, Fort Wayne, IN, United States; 4 Department of Family Medicine, University of Michigan, Ann Arbor, MI, United States; 5 Department of Epidemiology and Biostatistics, Michigan State University, East Lansing, MI, United States; 6 Division of Pediatric Endocrinology, University of Michigan, Ann Arbor, MI, United States; 7 Division of Endocrinology, Diabetes and Clinical Nutrition, Oregon Health and Science University Hospital, Portland, OR, United States; 8 Department of Neurology, University of Michigan, Ann Arbor, MI, United States; 9 Susan B. Meister Child Health Evaluation and Research Center, University of Michigan, Ann Arbor, MI, United States

**Keywords:** diabetes mellitus, glucose monitoring, continuous glucose monitoring, ecological momentary assessment, psychosocial factors, behavioral factors

## Abstract

**Background:**

Ecological momentary assessment (EMA) is a tool that captures emotional states, experiences, and behaviors in real or near-real time. Concurrent EMA and continuous glucose monitoring (CGM) data can provide an in-depth understanding of the impacts of psychosocial factors on momentary glucose levels. However, study methodology varies widely for EMA and CGM data collection and analysis.

**Objective:**

This scoping review aims to summarize the objectives, methodologies, and outcomes of studies analyzing concurrent biopsychosocial EMA and CGM data in diabetes.

**Methods:**

This study was conducted following the PRISMA-ScR (Preferred Reporting Items for Systematic Reviews and Meta-Analyses extension for Scoping Reviews) guidelines. Original research studies evaluating participants with diabetes, with concurrent collection and analysis of EMA and CGM data, were included in this review. A total of 782 studies were identified from PubMed, Embase, and EBSCOhost from May 2009 to December 2025. From these, duplicates, non-original research articles, and articles not measuring concurrent EMA and CGM data were excluded, resulting in a total of 19 studies included in this review. Methodological data were abstracted and summarized, including study characteristics, EMA protocols and outcomes, CGM outcomes, and integrated EMA and CGM study objectives. Methodological gaps across the included studies were identified and summarized.

**Results:**

Of the 19 studies, 16 primarily recruited adult populations, and the majority of these studies (n=14, 74%) included participants with type 1 diabetes. The median EMA delivery duration was 14 days (range 3‐18 days), with a median of 5.5 prompts delivered per day (range 1‐28). EMA outcomes included a variety of biopsychosocial factors (emotion, self-care behaviors, interpersonal interactions, symptoms, and cognition). Seventy-four percent analyzed blinded CGM data over a median of 14 (range 3‐18) days. Forty-two percent used nonstandardized CGM glucose outcomes (postprandial glucose, eating patterns, and hypoglycemic events). Integrated EMA and CGM data analysis answered a broad array of study objectives, including the impact of psychosocial factors on momentary glucose metrics; the influence of momentary glucose on emotional states, mood, personal behaviors, sleep, and cognition, or vice versa; and study protocol or mobile app optimization, among others. Methodological gaps included lack of standardization of EMA and CGM outcomes, duration of data capture, reporting of statistical analyses, and study population.

**Conclusions:**

This review provides a detailed summary of the methodology and outcomes of original research articles using combined EMA and blood glucose data measured via CGM. These combined methods allow for an opportunity to elucidate relationships between psychosocial factors and momentary glucose, an understanding of which is imperative for the development of future psychological and behavioral interventions in diabetes. However, standardization of protocols and expansion of participant populations for future EMA and CGM data collection and analysis are needed to ensure data accuracy, reproducibility, and generalizability.

## Introduction

Diabetes self-management is a dynamic process that involves various biological, psychological, social, and behavioral factors, including sleep, exercise, stress, and diet [1-4]. The complex system of influences and moment-to-moment changes in self-monitored blood glucose pose challenges to researching these interactions. Over the past two decades, diabetes technologies have expanded substantially with innovative methods to monitor and treat diabetes, as well as improve understanding of how biopsychosocial interactions influence the momentary changes in blood glucose.

Ecological momentary assessment (EMA) is an increasingly used tool that allows for the capture of momentary psychosocial factors, including individual experiences and behaviors in real time within the natural environment. EMA has become more widely available in research settings through the emergence of web-, phone-, and wearable health technology-based devices [5]. EMA improves reliability by decreasing recall bias, maximizing ecological validity, and gathering information about the micro processes that influence daily behaviors [5]. Compared to other longitudinal study methods, EMA allows for frequent, brief, and accessible assessments that provide an in-depth analysis of behaviors, experiences, and emotions at the individual level [6]. This makes EMA an ideal technology to use for patient-based research [5,7] on chronic diseases with multiple self-management tasks such as diabetes [8].

Self-monitoring of blood glucose (SMBG) is among the diabetes self-management tasks required daily for many individuals with diabetes. SMBG has historically been done with invasive and uncomfortable capillary glucose checks, which often results in inadequate glycemic data [9,10]. Continuous glucose monitoring (CGM) has dramatically changed SMBG by capturing glucose readings every 1‐15 minutes without the need for fingerstick [11], improving the SMBG experience [12-14], and illuminating the dynamic nature of momentary glucose patterns [15,16]. This large amount of glucose data has also highlighted the inadequacies of historically limited measures of glycemic control, such as glycated hemoglobin. Despite the widespread use of hemoglobin A_1c_ (HbA_1c_), analytical interferences and underlying medical conditions limit the accuracy of HbA_1c_ readings [17]. The granular glucose data obtained with CGM has allowed for more comprehensive CGM metrics that have now been standardized for clinical use and better describe the nuances found in momentary glucose fluctuations [18]. These CGM-derived “glucometrics” include the mean glucose, distributive measures such as the time above range (TAR; defined as glucose >180 mg/dL), time in range (TIR; defined as glucose 70‐180 mg/dL), time below range (TBR; defined as glucose <70 mg/dL), and measures of variability such as the coefficient of variation (CV) [18]. CGM allows researchers, clinicians, and individuals with diabetes to gain an understanding of how environmental, physiological, psychological, and behavioral influences impact glucose fluctuations [18].

Several reviews have examined the relationship between psychosocial factors and glucose. Mujis and colleagues [19] conducted a systematic review of 8 studies examining the association between glucose variability and mood in adults with diabetes. In this review, no clear association between glycemic variability and mood was found [19]. Another systematic review done by Nam et al [8] evaluated the relationships between psychosocial factors and diabetes self-management, finding that negative affect was associated with poor adherence to glucose monitoring and insulin injections, while stress was associated with binge eating behaviors. However, both reviews included studies that captured glucose using multiple modalities, including self-report and SMBG, which limits the availability and accuracy of glucose outcomes compared to the use of CGM.

Combining EMA and CGM methods provides a powerful opportunity to disentangle complex biopsychosocial influences and consequences of glycemic fluctuations in real or near-real time. Pairing these technologies can allow for higher validity in terms of patient responses [5] and can substantially lessen the amount of time between the measurements of glucose, psychological, and behavioral factors [20]. In a recent narrative review of EMA and CGM by Ehrmann et al [21], multiple research applications of these combined methodologies were noted, including a better understanding of the longitudinal variability in experiences and behaviors, delineation of objective versus subjective glycemia, and clarification of temporal associations between psychosocial factors and changes in glycemia. While these combined methods are increasingly used in research studies, Erhmann et al [21] also noted the potential for precision medicine clinical applications in the form of ecological momentary interventions such as just-in-time adaptive interventions.

Although a growing number of studies combining EMA and CGM data collected simultaneously are being conducted, there is a lack of consensus regarding the development of EMA protocols for question generation, delivery duration, delivery frequency, or delivery timing. Additionally, although CGM glucometrics have been standardized for clinical use, EMA-specific glucometrics have not been developed. Best practices regarding EMA-specific biopsychosocial outcomes and glucometrics are crucial to optimize the scientific rigor needed to ensure the validity and reliability of combined EMA and CGM studies, particularly if these data will be used for the development of future ecological momentary interventions. However, prior to the development of best practices, a more in-depth review of these studies is needed. Therefore, the main objective of this scoping review is to summarize the methods, outcomes, and results of studies that use both EMA and CGM data simultaneously as an initial step to standardizing future study protocols using these combined tools.

## Methods

### Protocol and Registration

The study protocol was developed in accordance with the PRISMA-ScR (Preferred Reporting Items for Systematic Reviews and Meta-Analyses extension for Scoping Reviews) statement [22,23] and can be found in Multimedia Appendix 1 and Checklist 1. This protocol was not registered.

### Eligibility Criteria

In line with the objective to summarize study methodologies of studies that analyzed concurrent EMA and CGM data in diabetes mellitus, studies were included if they were (1) peer-reviewed, original research studies; (2) recruited participants with diabetes; and (3) EMA and CGM data obtained and analyzed simultaneously. Studies were excluded if they (1) were non-original research articles (including exclusion of protocol papers, systematic reviews, meta-analyses, narrative reviews, or conference proceedings), (2) lacked CGM data or had asynchronous CGM data, (3) captured glycemic data using other modalities (including capillary, that is, fingerstick, glucoses or HbA_1c_), and (4) were studies not published in English.

### Information Sources

A literature review was performed using the PubMed, Elsevier Embase, and EBSCOhost CINAHL databases using the following key terms: “ecological momentary assessment AND blood glucose self-monitoring, diabetes mellitus, or glucose monitoring.” The search was conducted in May 2023 and again in July 2026, and articles published until December 2025 were considered for this review. Microsoft Excel version 2402 was used to organize and track the results during the review process.

### Search Strategy

The search strategy was developed in conjunction with a medical informationist and includes the following search terms: ecological momentary assessment, blood glucose self-monitoring, continuous glucose monitoring, blood sugar, CGM, self-monitor, and diabetes. The full search strategies for PubMed, Elsevier Embase, and EBSCOhost are shown in Multimedia Appendix 2.

### Selection of Sources of Evidence

The literature search resulted in a total of 782 studies from the 3 databases in 2 separate searches. After duplicates were removed, 610 studies remained. Two independent reviewers (KV and DA for the first search conducted in May 2023; KV and KM-S for the second search conducted in July 2026) reviewed study titles and abstracts of the 610 individual studies, and of these, 567 studies were excluded based on the prespecified exclusion criteria. Forty-three full-text studies were reviewed by two authors (KM-S and DA) independently; any disagreements were resolved by discussion with a third reviewer (KV), after which an additional 24 studies were excluded, for a total of 19 studies included in this review (as shown in the Results section).

### Data Charting Process

Key characteristics of EMA and CGM methodology were identified based on consensus from the 3 authors (KV, KM-S, and DA) prior to beginning the data extraction process. Included studies were reviewed, and details on study characteristics, EMA protocols, EMA outcomes, CGM characteristics, CGM outcomes, and integrated EMA and CGM data analysis and outcomes were extracted by 2 authors (KM-S and KV for the first search, KM-S and DA for the second search), charted in Microsoft Excel, and then discussed as a group of 3 reviewers (KM-S, KV, and DA) to resolve discrepancies. Throughout the data extraction process, additional study details were added to capture other pertinent methodological details found throughout multiple studies based on consensus from 3 authors (KV, KM-S, and DA). These additional study details included participant compensation, EMA response rates, and statistical analysis plans.

### Data Items

Study characteristics collected included study design (observational vs intervention), age range (target population age and sample age), diabetes type (type 1 and type 2), and sample size. EMA data collected included duration in days, prompts over the duration period (prompts per day, total responses over the study period), EMA response rate (percent completed), and psychological and/or behavioral outcomes as defined by each study. Similarly, CGM data included type of CGM device (blinded or personal), duration of wear, and glucose outcomes as defined by the study team (eg, mean glucose, TIR, TAR, TBR, SD, and CV). For integrated EMA and CGM data, study objectives, statistical methods, integrated EMA and CGM outcomes, and results were collected. A copy of this data extraction table is shown in Multimedia Appendix 3.

### Critical Appraisal of Individual Sources of Evidence

In line with the descriptive objective of this study to explore the broad study methodologies that incorporate both simultaneous EMA and CGM data, a critical appraisal of individual sources of evidence was not conducted.

### Synthesis of Results

A full descriptive summary of the study characteristics, EMA data, CGM data, and integration of EMA and CGM data are shown in the table in Multimedia Appendix 4. A summary of the current evidence from the included studies, as well as key methodological or study gaps, is highlighted in the gap map shown in the Results section.

## Results

### Selection of Sources of Evidence

As shown in Figure 1, a total of 19 studies were included in this review. Nonduplicate studies from the literature search were excluded primarily due to being a review, abstract, or protocol paper; not using CGM to collect blood glucose data; and not including study participants with diabetes.

**Figure 1.**
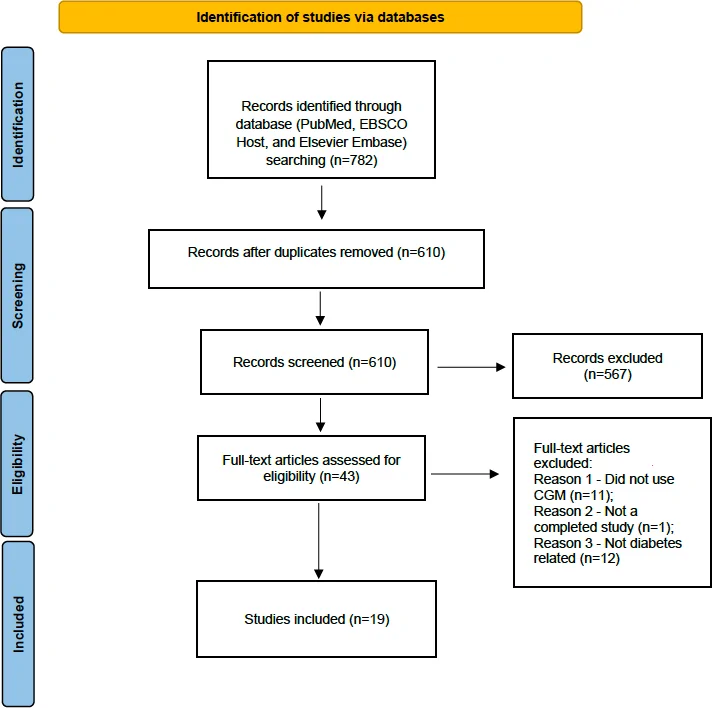
PRISMA-ScR (Preferred Reporting Items for Systematic Reviews and Meta-Analyses extension for Scoping Reviews) flow diagram. CGM: continuous glucose monitoring.

### Characteristics of Sources of Evidence

Study design characteristics across all studies are shown in Table 1. The majority of the studies were conducted in adult populations, while only 3 (19%) studies included adolescent populations. The weighted mean age of recruited participants was 44 years (range: 15.7‐61 years). Of the 19 studies, 14 (74%) were done in only type 1 diabetes, 2 studies were done in only type 2 diabetes, and 3 studies were done in both type 1 and type 2 diabetes populations. The sample sizes of the included studies varied widely from 18 to 602 participants (median 92, IQR 103). All studies were observational in nature.

**Table 1.** Characteristics of included studies.

First author, year	Target population	Population age range (years)	Age (years), mean (SD)^a^	Diabetes type	Sample size
Merwin, 2015 [24]	Adult	18‐68	42 (12)	Type 1	83
Moskovich, 2019 [25]	Adult	18‐65	42 (12)	Type 1	83
Ehrmann, 2022 [26]	Adult	18‐70	39 (13)	Type 1	200
Messer, 2022 [27]	Adolescent and adult	14‐26	18 (3)	Type 1	88
Hernandez, 2023 [28]	Adult	≥18	39.8 (14.4)	Type 1	92
Mascarenhas Fonseca, 2023 [29]	Adult	≥18	—^b^	Type 1	20
de Wit, 2023 [30]	Adult	≥18	44 (14)	Type 1	18
Soriano, 2023 [31]	Adult	51‐71	61 (10)	Type 2	63
Ehrmann, 2024 [32]	Adult	18‐70	T1DM^c^: 39 (13) T2DM^d^: 53 (10)	Type 1 and type 2	379
Hawks, 2024 [33]	Adult	18‐84	46 (16)	Type 1	200
Hernandez, 2024 [34]	Adult	≥18	40 (15)	Type 1	164
Merwin, 2024 [35]	Adult	18‐65	42 (12)	Type 1	83
Zaremba, 2024 [36]	Adult	45‐66	Median 56 (IQR 45‐66)	Type 1 and type 2	602
Gonzalez, 2025 [37]	Adult	≥18	40.6 (14.6)	Type 1	173
Gonzalez, 2025 [38]	Adult	≥18	40.1 (14.5)	Type 1	182
Hermanns, 2025 [39]	Adult	18‐70	T1DM: 39 (13) T2DM: 53 (9)	Type 1 and type 2	371
Horner, 2025 [40]	Adolescent	—^b^	15.7 (0.8)	Type 1	88
Saito, 2025 [41]	Adult	20‐69	T2DM: 53.1 (9.6) Control: 49.5 (8.2)	Type 2	36 (20 with T2DM)
Smith, 2025 [42]	Adolescent and young adult	15‐25	19.5 (3.1)	Type 1	34

a Unless otherwise denoted.

b Not reported in the published manuscript.

c T1DM: type 1 diabetes mellitus.

d T2DM: type 2 diabetes mellitus.

### Critical Appraisal Within Sources of Evidence

A critical appraisal of the included sources of evidence was not conducted as the objective of this study was a descriptive review of all possible methodologies incorporating concordant EMA measures of psychological and behavioral outcomes with blood glucose data measured via CGM.

### EMA Psychological and Behavioral Outcomes

EMA delivery of psychological and behavioral assessments showed considerable variability across studies, as shown in Table 2. The median duration of EMA delivery was 14 (range 3‐18) days with a median of 5.5 (range 1-28) prompts delivered per day. The median EMA response rate was high at 90.2% (IQR 15.4%), with lower response rates in longer-duration EMA studies (range of 66% response for 15 days of EMA to 96% for EMA duration of 3 days). Compensation was inconsistently reported but varied substantially (range: total compensation US $0‐100; compensation per EMA prompt US $0.33‐2.80).

EMA outcomes were heterogeneous and included emotions, behaviors, cognition, symptoms, workload, and contextual factors. Emotional constructs such as affect, motivation, self-efficacy, engagement, distress, and mood were measured in 10 studies and included positive and negative affect, diabetes motivation and engagement, emotional and diabetes distress, mood states, and health-related quality of life [24-28,30,32,37,38,41]. Behavioral constructs were measured in 9 studies (activity, diabetes self-management, eating behaviors, and relationship and social interactions) [24,25,28,31,35,37,40-42]. Various cognitive tasks were measured in 2 studies [29,33]. Additional EMA outcomes included sleep, workload, impacts of hypoglycemia, and diabetes symptoms [30,34,36,39].

**Table 2.** EMA^a^ methodology.

First author, year	Delivery duration (days)	Delivery frequency (time daily)	Total number of prompts over study	Percentage of prompts completed (%)	Psychological and behavioral outcomes
Merwin, 2015 [24]	3	1‐2 times per hour (14 hours daily)	42‐84	96.5	Emotions, diabetes, motivation, diabetes engagement, goal attainment^b^
Moskovich, 2019 [25]	3	1‐2 times per hour (14 hours daily)	45‐90	96.0	Emotional distress, objective binge-eating
Ehrmann, 2022 [26]	17	Once	17	79.1	Daily diabetes distress
Messer, 2022 [27]	14	2	28	—^c^	Behaviors, motivation, and attitudes toward diabetes self-management
Hernandez, 2023 [28]	14	5‐6	84	91	Activity, mental health, health-related quality of life
Mascarenhas Fonseca, 2023 [29]	15	6	90	81.7‐85.7	Cognitive EMA tasks
de Wit, 2023 [30]	14	6	84	70	Mood states, sleep duration, sleep quality
Soriano, 2023 [31]	7	5	35	91.0	Response to partner involvement, diabetes self-care
Ehrmann, 2024 [32]	17	Once	17	—	Diabetes distress, perceived glycemia
Hawks, 2024 [33]	15	3	45	66	Cognitive tasks (processing speed, sustained attention, executive functioning)
Hernandez, 2024 [34]	14	5‐6	84	93	Whole-day workload, workload consequences, well-being
Merwin, 2024 [35]	3	1‐2 times per hour (14 hours daily)	42‐84	96.5	Disordered eating
Zaremba, 2024 [36]	3	3	201	91	Physical, psychological, and social impacts of hypoglycemia
Gonzalez, 2025 [37]	14	5‐6	70‐84	89.4	Diabetes self-efficacy, diabetes distress, diabetes self-management
Gonzalez, 2025 [38]	14	5‐6	70‐84	95	Diabetes distress
Hermanns, 2025 [39]	18	Once	18	71.6	Diabetes symptoms (physical, emotional, and behavioral)
Horner, 2025 [40]	8	8	64	—	Diabetes self-management, affect, social interactions
Saito, 2025 [41]	14	4 times daily scheduled and before/after meals	—	T2DM^d^: 75 Control: 1.1	Mood, appetite, meal type, occasion, meal companion, sleep duration, dietary lapses
Smith, 2025 [42]	5	4	20	84.5	Hunger, eating behaviors for weight loss or weight maintenance

a EMA: ecological momentary assessment.

b As part of mobile app assessments.

c Not reported in the published manuscript.

d T2DM: type 2 diabetes mellitus.

### Glucose Monitoring and Outcomes

As shown in Table 3, of the 19 studies, 14 (74%) used blinded CGM; of these, 5 studies [28,33,34,37,38] allowed participants to also wear their personal CGM devices as well. The rationale for blinding was only reported in 1 study [24], which was to reduce reactivity to glucose values. CGM devices were worn for a median of 14 days with a range of 3‐18 days, coordinated with EMA protocols. CGM outcomes included both standardized and nonstandardized glucose-related outcomes. Just over half of the studies (58%) used any combination of standardized CGM glucometrics [18] including mean glucose, time in ranges (TAR, TIR, and TBR), and measures of variability such as SD or CV. Three studies used nonstandardized glucose outcomes as surrogate measures for disordered eating behaviors including insulin restriction [24,35] and binge-eating [25,35]. Other outcomes evaluated included hypoglycemic events [29], nocturnal hypoglycemia [30], 2-hour postprandial glucose [41], and glycemic variability prior to weight-focused eating behaviors [42].

**Table 3.** Glucose monitoring methodology.

First author, year	Type of glucose monitoring	Duration of monitoring (days)	Glucose outcomes^a^
Merwin, 2015 [24]	Blinded CGM^b^	3	Mean glucose, % total time>180 mg/dL^c^
Moskovich, 2019 [25]	Blinded CGM, self-report	3	Postprandial (120-minute) glucose^c^
Ehrmann, 2022 [26]	Personal CGM	17	Mean glucose, TIR, TBR, TAR, CV
Messer, 2022 [27]	Personal CGM	14	Mean glucose, TIR
Hernandez, 2023 [28]	Blinded CGM^d^	14	TIR, TBR, TAR
Mascarenhas Fonseca, 2023 [29]	Blinded CGM	10‐20	Hypoglycemic events
de Wit, 2023 [30]	Blinded CGM	14	CV, nocturnal hypoglycemia
Soriano, 2023 [31]	Blinded CGM	7	Mean glucose, TIR, TBR, TAR, SD, CV
Ehrmann, 2024 [32]	Personal CGM	14	TBR, TAR, CV
Hawks, 2024 [33]	Blinded CGM^d^	20	Mean glucose, SD, CV, TIR, TBR, TAR
Hernandez, 2024 [34]	Blinded CGM^d^	14	TIR
Merwin, 2024 [35]	Blinded CGM	3	Mean glucose, % total time>180 mg/dL^c^
Zaremba, 2024 [36]	Blinded CGM	70	TIR, TBR, TAR
Gonzalez, 2025 [37]	Blinded CGM^d^	14	Mean glucose, TIR, TBR, TAR, SD, CV
Gonzalez, 2025 [38]	Blinded CGM^d^	14	Mean glucose, TIR, TBR, TAR, SD, CV
Hermanns, 2025 [39]	Personal CGM	26	Mean glucose in a 2-hour period prior to EMA^e^
Horner, 2025 [40]	Personal CGM	8	Mean glucose, TIR, TBR, TAR, SD, CV
Saito, 2025 [41]	Blinded CGM	14	Mean glucose, TIR, 2-hour postprandial glucose
Smith, 2025 [42]	Blinded CGM	5	CV, SD in 4 hours prior to EMA prompt

a CV: coefficient of variation; TAR: time above range; TBR: time below range; TIR: time in range.

b CGM: continuous glucose monitoring.

c Surrogate outcomes to support insulin restriction.

d Participants able to wear personal CGM.

e EMA: ecological momentary assessment.

### Integrated EMA and CGM Data

A unique feature of the studies included in this review is the integration of EMA and glucose data measured via CGM (see Table 4). The purpose of integrating EMA and CGM data varied widely. Of the 19 studies, 8 (42%) evaluated the impacts of biopsychosocial factors, including psychological states [26,27,30,37,38,40,41], and workload [34] on CGM-derived glycemic outcomes or self-management behaviors. In contrast, 3 studies also evaluated the impact of CGM-derived glycemic outcomes on EMA-measured emotional states (such as diabetes distress [32,38]), mood [30], sleep [30], and cognition [33], recognizing that bidirectional associations were also possible. In the only 2 studies evaluating type 2 diabetes mellitus (T2DM) specific populations, both evaluated psychosocial domains in relation to glycemia. In the first study, integrated EMA and blinded CGM data were used to evaluate the effects of daily diabetes-specific social support from partners on daily glycemic outcomes, finding that higher levels of partner involvement improve short-term glycemic outcomes [31]. In the second study, the impact of psychosocial factors on eating behavior and CGM-derived glycemic outcomes was assessed, finding that stress led to dietary lapses with resulting higher daily glucose levels [41]. Similarly, in the one adolescent-only study, the effects of social interactions were evaluated on CGM-derived glycemic outcomes, finding that more social interaction improved TIR but also increased variability as measured by SD [40].

Of the 19 studies, 4 used integrated EMA and CGM data to evaluate disordered eating behaviors. In one study, CGM-derived glycemic variability was used to predict disordered eating behaviors measured via EMA [42]. In the other 3 studies, EMA-derived measures of negative affect were evaluated for correlations with either insulin restriction [24] or binge-eating behaviors [25] based on study-specific metrics from blinded CGM, and disordered eating profiles were developed [35].

Finally, investigators also used integrated EMA and CGM data for study protocol [29] or mobile app [36] optimization. In the study by Mascarenhas Fonseca et al [29], EMA-derived cognitive measures were delivered at low frequency/longer duration versus high frequency/shorter duration to determine which prompt period was superior in finding the most blinded CGM-derived hypoglycemic events, finding that low frequency/longer duration prompts were associated with a higher capture rate for hypoglycemic events [29]. In terms of the statistical analysis approach, due to the inherent repeated measures within EMA studies, most studies used mixed models for analysis.

**Table 4.** Integrated EMA^a^ and glucose outcomes and results.

First author, year	Study objective	Statistical analysis	EMA and glucose correlations found	EMA and glucose integrated outcomes^b^
Merwin, 2015 [24]	Effects of negative affect on insulin restriction.	Multilevel modeling	Yes	Negative affect before eating significantly associated with insulin restriction.
Moskovich, 2019 [25]	Impact of objective binge-eating on postprandial glucose.	Linear mixed models	Yes	Objective binge-eating associated with higher postprandial glucoses.
Ehrmann, 2022 [26]	Evaluate associations between diabetes distress and glycemia	Multilevel modeling	Yes	Higher daily distress significantly associated with daily TAR, TBR, and CV.
Messer, 2022 [27]	Understand which biopsychosocial factors predict glycemia.	Lasso, mixed models	Yes	Better sleep duration and quality, higher levels of motivation, and more positive attitudes associated with higher TIR.
Hernandez, 2023 [28]	Examine relationships between activity engagement and health-related quality of life in type 1 diabetes.	Multilevel modeling	Yes	Sleeping associated with lower glucoses. Caregiving and napping associated with higher glucoses.
Mascarenhas Fonseca, 2023 [29]	Determine if low frequency/longer duration EMA versus high frequency/shorter duration EMA captures more hypoglycemic events.	Paired *t* tests	Yes	Lower frequency/longer duration EMA associated with more hypoglycemic events.
de Wit, 2023 [30]	Determine (1) associations between glucose variability and mood; (2) associations between hypoglycemia and sleep duration and quality.	Mixed models	No	No relationships found between glucose variability and mood fluctuations.
Soriano, 2023 [31]	Evaluate associations between partner involvement in diabetes self-management and glycemia.	Dynamic structural equation modeling	Yes	Associations found in the following glucometrics *preceding* higher partner involvement: higher mean glucose and higher % in target TBR. Associations also found in these glucometrics *following* higher partner involvement: lower mean glucose level, higher % in target TIR, higher % in target TAR, lower SD, and lower CV.
Ehrmann, 2024 [32]	Determine if objective-CGM^c^ glycemia or subjective perceptions of glycemia are more predictive of diabetes distress.	Bayesian linear mixed effects regression	Yes	Perceived glucose variability and perceived hyperglycemia are more strongly associated with daily distress than objective measures of hyperglycemia and glycemic variability on CGM.
Hawks, 2024 [33]	Evaluate associations between cognition and glucose and to identify individual differences in cognitive vulnerability to glucose fluctuations.	Hierarchical Bayesian modeling; Lasso-driven regression	Yes	Large glucose fluctuations were associated with slower and less accurate processing speed; small glucose elevations were associated with faster processing speed.
Hernandez, 2024 [34]	Examine reliability and validity of using the NASA Task Load Index to assess whole-day workload; correlations between workload and glycemia.	Multilevel modeling	Yes	Higher whole-day workload was associated with lower TIR.
Merwin, 2024 [35]	Examine differences in disordered eating behavior and glycemic outcome profiles.	ANOVA; Levene test with bootstrapping; Games-Howell bootstrapped multiple comparisons tests	Yes	“Bulimia” and “binge eating” groups both were higher than the “overeating” and “low pathology” groups.
Zaremba, 2024 [36]	Determine factors associated with app (which measured physical, psychological, and social impacts of hypoglycemia).	Linear regression with Bonferroni correction	Yes	Higher TBR (level II) was associated with higher EMA completion rates.
Gonzalez, 2025 [37]	Analyze relationships between self-efficacy, diabetes distress, self-management behaviors, and glycemic regulation.	Dynamic structural equation modeling	Yes	Morning self-efficacy predicted lower diabetes distress and better self-management, which in turn improves glycemic regulation. Prior day self-efficacy, diabetes distress, and self-management correlated to next morning self-efficacy.
Gonzalez, 2025 [38]	Evaluate bidirectional correlations between diabetes distress and glycemic regulation.	Multilevel cross-lagged panel modeling	Yes	Higher mean glucose, lower TIR, higher TAR, and higher CV predicted higher levels of diabetes distress. Higher diabetes distress predicted lower TBR.
Hermanns, 2025 [39]	Evaluate potential drivers of diabetes symptoms.	Linear mixed effects models	Yes	Four symptoms (speech difficulties, coordination problems, confusion, and food cravings) showed higher symptom intensity with lower glucose levels. Four symptoms (increased thirst, urge to urinate, itching, and taste disturbance) showed higher symptom intensity with higher glucose levels.
Horner, 2025 [40]	Assess how diabetes self-care behaviors, mood, and social interactions influence subsequent glucose outcomes.	Dynamic structural equation modeling	Yes	Diabetes self-care predicted lower mean glucose, TIR, and TAR. Social interactions increased SD, TIR, and TAR.
Saito, 2025 [41]	Assess psychological factors affecting daily glucose and TIR.	Univariate logistic regression followed by multilevel modeling	Yes	Higher daily stress and dietary lapses associated with higher daily glucose and TAR. Longer sleep duration associated with lower daily glucose, higher TIR, and lower TAR.
Smith, 2025 [42]	Evaluate whether glycemic variability predicts hunger or disordered eating behaviors.	Linear and generalized linear mixed models	Yes	Higher glycemic variability was correlated with eating behaviors for weight loss/weight maintenance.

a EMA: ecological momentary assessment.

b CV: coefficient of variation; TAR: time above range; TBR: time below range; TIR: time in range.

c CGM: continuous glucose monitoring.

### Methodological Gaps in Studies With Combined EMA and CGM Data

Despite the increased use of studies combining EMA and CGM quantitative data sources, there is little published guidance on protocol development. A review of the existing literature demonstrates notable methodological gaps, which are highlighted in Table 5. While one study evaluated the duration of EMA protocols specifically for future study optimization [29], consensus regarding the duration of EMA data collection and EMA question prompt frequency to minimize response burden and maximize response validity has not been reached. Additionally, there are very few EMA-specific outcome measures, and the psychometric properties of EMA questions derived from previously validated patient-response outcome measures used in the included studies were either not fully evaluated or not reported on by study teams.

**Table 5.** Methodological gap map for studies combining EMA^a^ and CGM^b^ data.

Methodological domain	Current evidence	Methodological gaps
EMA duration	Mostly 3‐18 days	No consensus on ideal duration
EMA frequency	1‐14 prompts/day	No consensus on ideal frequency No resolution on participant burden versus data granularity Limited assessment of response fatigue and impact on data validity
EMA response rate	Typically higher than 80%	Missingness may be nonrandom
EMA item validation	Many items adapted from validated scales	Very few EMA-specific outcome measures Limited psychometric properties of adapted patient-reported outcome measures for EMA
CGM type	Majority were blinded CGM	Limited reporting rationale for blinded versus unblinded CGM
CGM duration	Mostly 14 days (range 3‐70 days)	Limited reporting of rationale for CGM duration Inconsistent reporting of CGM wear time Inadequate reporting of missing data
CGM outcomes	Highly variable (slightly over half used standardized CGM glucometrics)	Variable CGM duration used for standard CGM glucometrics Potential misalignment between standardized CGM glucometrics and momentary nature of EMA variables Limited validity of nonstandardized CGM metrics
Statistical methods	Primarily mixed models or advanced repeated-measures models	Limited comparability across studies
Causality	All studies were observational	Need longitudinal and intervention studies
Population diversity	Primarily adults with T1DM^c^	Children, adolescents, older adults, and individuals with T2DM^d^ are underrepresented

a EMA: ecological momentary assessment.

b CGM: continuous glucose monitoring.

c T1DM: type 1 diabetes mellitus.

d T2DM: type 2 diabetes mellitus.

Similarly, methodological gaps exist in CGM data collection as well. The majority of studies used blinded CGM for data collection though the rationale for CGM type was only reported in one study [24]. Additionally, over half of the studies (58%) used standardized CGM outcome measures though the duration of CGM data collection varied widely, potentially limiting the accuracy of these outcomes as standard duration for CGM data collection is 14 days [43]. The studies that used nonstandardized CGM outcomes may have been more specifically tailored to the momentary nature of the EMA data though the validity of these outcome measures is unclear.

The largest methodological design gaps involve the studied populations. Combined EMA and CGM studies have been primarily done in adults with type 1 diabetes mellitus (T1DM). Additionally, only 3 of the studies included adolescent populations and none of the studies specifically focused on older adults with diabetes. Whether or not associations found generalize to other populations such as individuals with T2DM or across the lifespan are not clear based on the available evidence.

## Discussion

### Summary of the Evidence

This review summarizes 19 studies that correlate EMA-measured psychosocial, cognitive, or behavioral factors with CGM-derived glucose data. Although the included studies primarily focused on adults with T1DM, other study design characteristics varied widely including sample size, EMA delivery duration, EMA frequency, and EMA biopsychosocial outcomes. Most of the studies captured glucose using participant-blinded data though CGM outcomes were nearly equally divided into either standard CGM glucometric outcomes or nonstandardized outcomes.

Study objectives varied widely but included the impacts of psychological factors on momentary glucose; the effects of momentary glucose on emotional states, mood, personal behaviors, sleep, and cognition; and optimization of study protocols or mobile apps. Advanced statistical models were used in nearly all studies to integrate EMA and glucose data.

Despite the wide variability in EMA protocols including the duration and frequency of prompts, the included studies overall demonstrated high levels of engagement with EMA. Mirroring the findings of one of the included reviews by Mascarenhas Fonseca et al [29], higher completion rates were seen in studies with shorter EMA duration versus longer EMA duration, perhaps reflective of higher participant burden. Based upon this review, EMA prompts may be optimized with durations between 3 and 7 days, which is an important consideration to reduce missing data and the resulting self-selection bias that has been found in EMA data [44]. However, Masacarenhas Fonesca et al [29] also found that although shorter EMA duration with higher frequency prompts led to higher overall completion rates, longer EMA duration with lower frequency prompts captured more hypoglycemia events, which was the primary glycemic outcome in this study. This suggests that different combinations of EMA duration and prompt frequency may be appropriate depending on the study purpose but should be evaluated and reported by study teams.

The biopsychosocial outcome measures varied broadly, as was shown in prior reviews of combined EMA and CGM data [21]. Although the majority of these studies developed EMA questions based on validated patient-reported outcome measures, these question items have not been validated for use in EMA protocols. As Ehrmann et al [21] noted in their review, standardization of EMA-item development, validation, and response cutoffs that are clinically meaningful is needed to ensure the scientific rigor for determining accurate associations between biopsychosocial factors and glucose prior to use in precision medicine applications such as ecological momentary interventions [21].

There was also significant heterogeneity in CGM duration and CGM outcomes measured in the studies included in this review. Slightly over half of the included studies used clinically standardized CGM outcome measures. According to the International Consensus on Time in Range [18], 14 days of CGM use correlates the strongest with 3-month data for key glucometrics [45,46] and is now the standard duration on the ambulatory glucose profile from CGM downloads [47]. Analysis of timeframes less than 14 days for standardized CGM glucometric outcomes, which was done in 5 studies [26,31,33,36,40], may result in glycemic data that is less representative for that individual. On the other hand, analysis of 14-day glucose data may not be in line with the momentary nature of EMA data collection. The remaining studies used nonstandardized glucose outcomes, which may better reflect the momentary nature of the study question but have limited validity. The broad range of glucose metrics, particularly nonstandardized metrics, also creates challenges with translating results to direct clinical care such as associations with longer-term diabetes outcomes such as retinopathy [48]. Validation of EMA-specific CGM metrics is important to ensure these outcomes are clinically meaningful, particularly as individuals without diabetes can experience glucose elevations detected on CGM that do not have any clinically important consequences [49].

### Limitations

Although this review provides a comprehensive overview of the methods, outcomes, and results of original research studies that used both EMA and CGM data simultaneously in participants with diabetes, there are a number of limitations. First, although the EMA and CGM outcomes varied widely, the populations that these studies applied to focused primarily on adults. Therefore, gaps in using combined EMA and CGM methods for research remain in children, adolescents, and older populations. EMA may be challenging with children due to lack of access to digital health technologies [50] and ethical, cognitive, and developmental considerations for directly assessing child behavior and emotional states [51]. Although there is some evidence that EMA may be successfully implemented in children as young as 7 years [52], these studies have not been used in conjunction with CGM. The lack of EMA studies in older adult populations may be reflective of lower technology acceptance or confidence in this population. Previous literature has shown that feelings of inadequacy, anxiety, and overall lack of exposure surpass older participants’ willingness to adopt new technologies [53-55]. In addition, all but one of the included studies enrolled individuals with T1DM (92%) and only 4 studies (31%) enrolled participants with T2DM. This may be due to the need for frequent SMBG, which is recommended for all individuals with T1DM due to the need for insulin therapy. In contrast, only about one-quarter of individuals with T2DM require insulin, which is the main indication for SMBG in T2DM [56]. Future studies may benefit from expanding the populations enrolled to include a broader participant pool to ensure associations between psychosocial factors and glucose are generalizable.

Another limitation is the small sample size of original research studies using both EMA and CGM data together in one study design. Due to this, a meta-analysis was not feasible. Additionally, the significant heterogeneity in study design limits conclusions that can be definitively drawn for ideal EMA protocol design recommendations such as biopsychosocial question development and the duration and timing of question delivery, as well as CGM glucose outcomes. However, nearly all of the included studies were published just in the last 5 years, highlighting an increased use of these combined methodologies and highlighting the urgent need for standardizing EMA-specific biopsychosocial outcomes and EMA-specific CGM outcomes to ensure validity and reliability of this data. Nevertheless, this review provides a very detailed overview of the methodology and outcomes of original research studies that combine biopsychosocial EMA and CGM data.

### Conclusions

Together, EMA and CGM can be used to disentangle the complex interactions between biopsychosocial factors and glucose data. However, given the broad application of these methods and lack of associated clinical relevance, best practices surrounding study design regarding biopsychosocial EMA protocols and CGM metrics are urgently needed to ensure scientific rigor and accuracy. This review provides a comprehensive overview of the studies using combined EMA and CGM methods in diabetes, which is an important stepping stone in the development of these best practices.

## Supplementary material

10.2196/87688 Multimedia Appendix 1 Ecological momentary assessment (EMA) and continuous glucose monitoring (CGM) scoping review protocol.

10.2196/87688 Multimedia Appendix 2 Search strategy.

10.2196/87688 Multimedia Appendix 3 Data extraction table.

10.2196/87688 Multimedia Appendix 4 Synthesis of results.

10.2196/87688 Checklist 1 PRISMA-ScR checklist.

## Abbreviations

CGM: continuous glucose monitoring
CV: coefficient of variation
EMA: ecological momentary assessment
HbA_1c_: hemoglobin A_1c_
PRISMA-ScR: Preferred Reporting Items for Systematic Reviews and Meta-Analyses extension for Scoping Reviews
SMBG: self-monitoring of blood glucose
T1DM: type 1 diabetes mellitus
T2DM: type 2 diabetes mellitus
TAR: time above range
TBR: time below range
TIR: time in range

## References

[1] Tiwari R, Tam DNH, Shah J, Moriyama M, Varney J, Huy NT (2021). Effects of sleep intervention on glucose control: a narrative review of clinical evidence. Prim Care Diabetes.

[2] Guelfi KJ, Ratnam N, Smythe GA, Jones TW, Fournier PA (2007). Effect of intermittent high-intensity compared with continuous moderate exercise on glucose production and utilization in individuals with type 1 diabetes. Am J Physiol Endocrinol Metab.

[3] Falco G, Pirro PS, Castellano E, Anfossi M, Borretta G, Gianotti L (2015). The relationship between stress and diabetes mellitus. J Neurol Psychol.

[4] Henry CJ, Kaur B, Quek RYC (2020). Chrononutrition in the management of diabetes. Nutr Diabetes.

[5] Shiffman S, Stone AA, Hufford MR (2008). Ecological momentary assessment. Annu Rev Clin Psychol.

[6] Warnick JL, Westen SC, Albanese-O’Neill A (2020). Use of ecological momentary assessment to measure self-monitoring of blood glucose adherence in youth with type 1 diabetes. Diabetes Spectr.

[7] Stone AA, Shiffman S, Atienza A, Shiffman S, Nebeling L (2007). The Science of Real-Time Data Capture: Self-Reports in Health Research.

[8] Nam S, Griggs S, Ash GI (2021). Ecological momentary assessment for health behaviors and contextual factors in persons with diabetes: a systematic review. Diabetes Res Clin Pract.

[9] Wagner J, Malchoff C, Abbott G (2005). Invasiveness as a barrier to self-monitoring of blood glucose in diabetes. Diabetes Technol Ther.

[10] Ong WM, Chua SS, Ng CJ (2014). Barriers and facilitators to self-monitoring of blood glucose in people with type 2 diabetes using insulin: a qualitative study. Patient Prefer Adherence.

[11] Ray MK, McMichael A, Rivera-Santana M, Noel J, Hershey T (2021). Technological ecological momentary assessment tools to study type 1 diabetes in youth: viewpoint of methodologies. JMIR Diabetes.

[12] Polonsky WH, Hessler D (2013). What are the quality of life-related benefits and losses associated with real-time continuous glucose monitoring? A survey of current users. Diabetes Technol Ther.

[13] Polonsky WH, Hessler D, Ruedy KJ, Beck RW, Group DS (2017). The impact of continuous glucose monitoring on markers of quality of life in adults with type 1 diabetes: further findings from the DIAMOND randomized clinical trial. Diabetes Care.

[14] Gilbert TR, Noar A, Blalock O, Polonsky WH (2021). Change in hemoglobin A1c and quality of life with real-time continuous glucose monitoring use by people with insulin-treated diabetes in the Landmark study. Diabetes Technol Ther.

[15] Ajjan RA (2017). How can we realize the clinical benefits of continuous glucose monitoring?. Diabetes Technol Ther.

[16] Choudhary P, Olsen BS, Conget I, Welsh JB, Vorrink L, Shin JJ (2016). Hypoglycemia prevention and user acceptance of an insulin pump system with predictive low glucose management. Diabetes Technol Ther.

[17] Lundholm MD, Emanuele MA, Ashraf A, Nadeem S (2020). Applications and pitfalls of hemoglobin A1C and alternative methods of glycemic monitoring. J Diabetes Complications.

[18] Battelino T, Danne T, Bergenstal RM (2019). Clinical targets for continuous glucose monitoring data interpretation: recommendations from the international consensus on time in range. Diabetes Care.

[19] Muijs LT, Racca C, de Wit M (2021). Glucose variability and mood in adults with diabetes: a systematic review. Endocrinol Diabetes Metab.

[20] Rethorst CD, Githinji P, Seguin-Fowler RA, MacMillan Uribe AL, Szeszulski J, Liao Y (2023). Real-time assessment of the bidirectional relationship between affective states and glucose: protocol for a 14-day observational study. JMIR Res Protoc.

[21] Ehrmann D, Hermanns N, Schmitt A, Klinker LY, Kulzer B (2026). Ecological momentary assessment of person-reported outcomes in diabetes: unlocking insights with continuous glucose monitoring and the potential for precision medicine. Curr Diab Rep.

[22] Page MJ, McKenzie JE, Bossuyt PM (2021). The PRISMA 2020 statement: an updated guideline for reporting systematic reviews. BMJ.

[23] Tricco AC, Lillie E, Zarin W (2018). PRISMA Extension for Scoping Reviews (PRISMA-ScR): checklist and explanation. Ann Intern Med.

[24] Merwin RM, Dmitrieva NO, Honeycutt LK (2015). Momentary predictors of insulin restriction among adults with type 1 diabetes and eating disorder symptomatology. Diabetes Care.

[25] Moskovich AA, Dmitrieva NO, Babyak MA (2019). Real-time predictors and consequences of binge eating among adults with type 1 diabetes. J Eat Disord.

[26] Ehrmann D, Schmitt A, Priesterroth L, Kulzer B, Haak T, Hermanns N (2022). Time with diabetes distress and glycemia-specific distress: new patient-reported outcome measures for the psychosocial burden of diabetes using ecological momentary assessment in an observational study. Diabetes Care.

[27] Messer LH, Vigers T, Pyle L (2022). Novel predictors of daily fluctuations in glycemia and self-management in adolescents and young adults with type 1 diabetes. Diabet Med.

[28] Hernandez R, Schneider S, Pham L, Pyatak EA (2023). Across and within-individual associations between everyday activities and quality of life relevant measures, in workers with type 1 diabetes. Appl Res Qual Life.

[29] Mascarenhas Fonseca L, Strong RW, Singh S (2023). Glycemic variability and fluctuations in cognitive status in adults with type 1 diabetes (GluCog): observational study using ecological momentary assessment of cognition. JMIR Diabetes.

[30] de Wit M, van Raalte DH, van den Berg K (2023). Glucose variability and mood in people with type 1 diabetes using ecological momentary assessment. J Psychosom Res.

[31] Soriano EC, Polonsky WH (2023). The influence of real-time continuous glucose monitoring on psychosocial outcomes in insulin-using type 2 diabetes. J Diabetes Sci Technol.

[32] Ehrmann D, Hermanns N, Schmitt A, Klinker L, Haak T, Kulzer B (2024). Perceived glucose levels matter more than CGM-based data in predicting diabetes distress in type 1 or type 2 diabetes: a precision mental health approach using n-of-1 analyses. Diabetologia.

[33] Hawks ZW, Beck ED, Jung L (2024). Dynamic associations between glucose and ecological momentary cognition in type 1 diabetes. NPJ Digit Med.

[34] Hernandez R, Schneider S, Jin H (2024). Whole day workload: evaluation of a new outcome measure in occupational therapy for adults with type 1 diabetes. Am J Occup Ther.

[35] Merwin RM, Dmitrieva NO, Moskovich AA (2024). Profiles of disordered eating behaviour in type 1 diabetes using the DEPS-R and behaviour and glycaemic outcomes in a real-life setting. Diabet Med.

[36] Zaremba N, Martine-Edith G, Divilly P (2024). Associations of clinical, psychological, and sociodemographic characteristics and ecological momentary assessment completion in the 10-week Hypo-METRICS study: hypoglycaemia measurements thresholds and impacts. Diabet Med.

[37] Gonzalez JS, Schneider S, Hoogendoorn C, Nandoo L, Pyatak E (2025). Self-efficacy, diabetes distress, self-management, and glycemic regulation: within-person pathways in type 1 diabetes. Ann Behav Med.

[38] Gonzalez JS, Hoogendoorn CJ, Hernandez R (2025). Diabetes-related distress and glycemic dysregulation in everyday life with type 1 diabetes: which comes first?. Diabetes Care.

[39] Hermanns N, Ehrmann D, Kulzer B, Klinker L, Haak T, Schmitt A (2025). Somatic and mental symptoms associated with dysglycaemia, diabetes-related complications and mental conditions in people with diabetes: assessments in daily life using continuous glucose monitoring and ecological momentary assessment. Diabetes Obes Metab.

[40] Horner FS, Helgeson VS (2025). Glucose in the dynamic psychosocial environment: findings from adolescents with type 1 diabetes. Biopsychosoc Sci Med.

[41] Saito J, Kumano H (2024). Psychosocial factors influencing dietary management in patients with type 2 diabetes and healthy adults: an ecological momentary assessment approach. Front Psychol.

[42] Smith LB, Ahlich E, Lang B (2025). Glycemic variability and weight-focused eating behaviors among adolescents and young adults with type 1 diabetes. J Pediatr Psychol.

[43] Ajjan RA, Battelino T, Cos X (2024). Continuous glucose monitoring for the routine care of type 2 diabetes mellitus. Nat Rev Endocrinol.

[44] Drexl K, Ralisa V, Rosselet-Amoussou J (2025). Readdressing the ongoing challenge of missing data in youth ecological momentary assessment studies: meta-analysis update. J Med Internet Res.

[45] Xing D, Kollman C, Beck RW (2011). Optimal sampling intervals to assess long-term glycemic control using continuous glucose monitoring. Diabetes Technol Ther.

[46] Riddlesworth TD, Beck RW, Gal RL (2018). Optimal sampling duration for continuous glucose monitoring to determine long-term glycemic control. Diabetes Technol Ther.

[47] American Diabetes Association Professional Practice Committee (2024). 6. Glycemic goals and hypoglycemia: *Standards of Care in Diabetes—2024*. Diabetes Care.

[48] Lachin JM, Bebu I, Gao X, Nathan DM, Zinman B, Diabetes C (2022). Association of estimated time-in-range capillary glucose levels versus HbA1c with progression of microvascular complications in the diabetes control and complications trial. Diabetes Care.

[49] Avner S, Robbins T (2025). A scoping review of glucose spikes in people without diabetes: comparing insights from grey literature and medical research. Clin Med Insights Endocrinol Diabetes.

[50] Danet M (2020). Parental concerns about their school-aged children’s use of digital devices. J Child Fam Stud.

[51] Mantilla A, Edwards S (2019). Digital technology use by and with young children: a systematic review for the Statement on Young Children and Digital Technologies. Australas J Early Child.

[52] Heron KE, Everhart RS, McHale SM, Smyth JM (2017). Using mobile-technology-based ecological momentary assessment (EMA) methods with youth: a systematic review and recommendations. J Pediatr Psychol.

[53] Heinz M, Martin P, Margrett JA (2013). Perceptions of technology among older adults. J Gerontol Nurs.

[54] Mitzner TL, Boron JB, Fausset CB (2010). Older adults talk technology: technology usage and attitudes. Comput Human Behav.

[55] Vaportzis E, Clausen MG, Gow AJ (2017). Older adults perceptions of technology and barriers to interacting with tablet computers: a focus group study. Front Psychol.

[56] Pitak P, Tasai S, Kumpat N (2023). The prevalence of glycemic control in patients with type 2 diabetes treated with insulin: a systematic review and meta-analysis. Public Health.

