# Supplementary material for: Real-Time Psychological Factors Using Ecological Momentary Assessment With Continuous Glucose Monitoring in Diabetes Mellitus: Scoping Review
Source: JMIR Diabetes. 2026 Sep 30;11:e87688. doi: 10.2196/87688 (PMC13626547; doi:10.2196/87688)
Supplement: Supplementary file 2 [file diabetes-v11-e87688-s001.docx]

**Multimedia Appendix 1: EMA and CGM Scoping Review Protocol**

**Introduction:**

Measurement of momentary biopsychosocial factors measured via EMA combined with momentary glucose data collected via CGM have significant potential to allow for an in-depth understanding of these associations. This understanding is the crucial for the development and evaluation of psychologic or behavioral interventions. Standardization of EMA and CGM data collection can contribute to accuracy and reproducibility of the collected data. Prior to standardization, the broad spectrum of studies with simultaneous EMA and CGM data must first be summarized.

**Objective:**

To identify, evaluate and summarize studies that evaluate psychological and/or behavioral characteristics using ecological momentary assessment (EMA) methodology in the context of glucose data measured via continuous glucose monitoring (CGM).

**Inclusion Criteria:**

- Participants: Study participants must have diabetes
- Studies: EMA data must be collected simultaneously with glycemic data measured via CGM. EMA data and CGM data must also be analyzed together in the same statistical models. Only studies published in English will be included in this review.

**Exclusion Criteria:**

- Studies: Non-original research studies (including protocol papers, systematic reviews, meta-analyses, narrative reviews, or conference proceedings) will be excluded. Additionally, blood glucose data needs to be obtained via CGM collection and not via capillary glucose or glycated hemoglobin. Studies with discontinued or absent glucose data will be excluded.

**Methods:**

This study will be conducted in accordance with the Preferred Reporting Items for Systematic reviews and Meta-Analyses extension for Scoping Reviews.

*Literature Review:*

The following databases will be search for articles pertaining the aforementioned inclusion and exclusion criteria: PubMed, Elsevier Embase, and EBSCOhost CINAHL. No pre-specified “start” date will be included as part of the search strategy given that the mandatory include of CGM data was already a time-limited pre-specification as CGM devices have only been available since 2004. Due to the lack of funding for translation services, only articles published in English will be included.

*Search terms:*

In consultation with an Informationist at the School of Medicine Library, search terms will include: “ecological momentary,” “ecological momentary assessment”, “diabetes,” “continuous glucose monitoring,” “glucose metabolism disorders,” “blood sugar,” “CGM,” “blood glucose self-monitoring,” and “glucose self-monitor.”

*Study procedures:*

Following the initial literature search, duplicate studies will be removed. Two independent reviewers will read all the non-duplicated studies in the literature search to remove non-original research articles, studies not published in English, studies not including diabetes participants, studies not measuring glucose data using CGM and studies not measuring and/or analyzing EMA and CGM data concurrently. Any disagreements on excluded studies will be resolved by a third reviewer. PDF copies of all study titles and abstracts will be stored in the University of Michigan (UM) DropBox.

*Data extraction:*

Following determination of included studies in the scoping review, PDF copies of the studies will be stored in UM DropBox. Two reviewers will independently reach each study and collect the following variables for each study:

Study characteristics:

- Target population
- Age range target
- Mean age of recruited participants
- Diabetes Type
- Number of participants
- Compensation

EMA characteristics:

- EMA duration
- EMA delivery frequency
- Total number of prompts over the study period
- EMA completion rate
- EMA constructs measured
- EMA outcomes measured

CGM characteristics:

- Blinded or personal CGM
  - If blinded, was personal CGM allowed?
- Number of days measured
- Glucose outcomes
- Is the glucose a surrogate for a behavioral outcome?

Integrated EMA and CGM analysis characteristics:

- Study objective
- Were EMA and CGM correlations found?
- Type of statistical analysis
- Main findings

All data extracted will be organized using Microsoft Excel version and stored on UM DropBox. Following data extraction by 2 independent reviewers, any discrepancies will be resolved with a third reviewer.

*Data Analysis*

Following data extraction, we will determine if EMA or CGM measures (i.e. psychology or behavioral constructs) or procedures (i.e. EMA or CGM duration or frequency) are used are used in two or more different studies. If study measures and procedures are not similar amongst two or more different studies, data from all studies will be summarized in 5 tables: Study Characteristics, EMA Characteristics, CGM Characteristics, EMA/CGM Data Integration Characteristics, and Methodological Study Gap Map.
