# Supplementary material for: Real-Time Psychological Factors Using Ecological Momentary Assessment With Continuous Glucose Monitoring in Diabetes Mellitus: Scoping Review
Source: JMIR Diabetes. 2026 Sep 30;11:e87688. doi: 10.2196/87688 (PMC13626547; doi:10.2196/87688)
Supplement: Supplementary file 3 [file diabetes-v11-e87688-s002.docx]

**Multimedia Appendix 2: Search Strategy**

**NLM PubMed (210 results on July 23, 2026)**

(ecological momentary assessment[mh] OR "ecological momentary"[tiab:~2]) AND (blood glucose self-monitoring[mh] OR continuous glucose monitoring[mh] OR glucose metabolism disorders[mh] OR "blood sugar"[tiab] OR "blood sugars"[tiab] OR cgm[tiab] OR diabet*[tiab] OR glucose[tiab] OR self-monitor*[tiab])

**Elsevier Embase (343 results on July 23, 2026)**

('ecological momentary assessment'/exp OR (ecological NEAR/2 momentary):ti,ab) AND ('blood glucose monitoring'/exp OR 'diabetes mellitus'/exp OR (blood NEAR/2 sugar*):ti,ab OR cgm:ti,ab OR diabet*:ti,ab OR ((glucose OR self) NEAR/3 monitor*):ti,ab)

**EBSCOhost CINAHL (229 results on July 23, 2026)**

(TI "ecological momentary" OR AB "ecological momentary") AND (MH "diabetes mellitus+" OR MH "Blood Glucose Monitoring+" OR TI (blood N3 sugar*) OR AB (blood N3 sugar*) OR TI cgm OR AB cgm OR TI diabet* OR AB diabet* OR TI ((glucose OR self) N3 monitor*) OR AB ((glucose OR self) N3 monitor*))

**Updated Search July 23, 2026:**

**Total Articles from Each Database:** 782

*Before removal of duplicates:*

PubMed: 210

Embase: 343

EBSCO Host CINAHL: 229

*After removal of duplicates:*

Total: 610
