# Supplementary material for: Real-Time Psychological Factors Using Ecological Momentary Assessment With Continuous Glucose Monitoring in Diabetes Mellitus: Scoping Review
Source: JMIR Diabetes. 2026 Sep 30;11:e87688. doi: 10.2196/87688 (PMC13626547; doi:10.2196/87688)
Supplement: Supplementary file 4 [file diabetes-v11-e87688-s003.docx]

**Multimedia Appendix 3: Data Extraction Table**
